# Intratumoral and peritumoral radiomics for preoperative prediction of the efficacy of HIFU ablation for uterine fibroids based on multiparametric MRI: a multicenter study

**DOI:** 10.3389/fonc.2025.1699632

**Published:** 2026-01-13

**Authors:** Chengwei Li, Hongjian Liao, Jian Liu, Jin Gao

**Affiliations:** Department of Radiology, The Third People’s Hospital of Chengdu, Chengdu, China

**Keywords:** efficacy prediction, high-intensity focused ultrasound, magnetic resonance imaging, radiomics, uterine fibroids

## Abstract

**Objectives:**

To assess the predictive value of intratumoral and multiregion peritumoral radiomics based on multiparametric MRI for preoperatively predicting the efficacy of high-intensity focused ultrasound (HIFU) ablation of uterine fibroids.

**Materials and methods:**

This retrospective study included 360 patients with uterine fibroids treated with high-intensity focused ultrasound (HIFU) at Center A (training set: N = 240; internal testing set: N = 60) and Center B (external testing set: N = 60). Patients were grouped into sufficient or insufficient ablation categories based on postoperative non-perfusion volume ratio. Intratumoral regions (TRs) were manually delineated on T2-weighted imaging (T2WI) and contrast-enhanced T1-weighted imaging (CE-T1WI). Peritumoral regions (PTRs) were generated by expanding the tumor boundary by 1 mm, 3 mm, and 5 mm. Radiomics features were extracted from TRs and PTRs on both MRI sequences. Key features for preoperative prediction were selected using t-tests, Pearson correlation, and LASSO regression. Support vector machine (SVM) models were built for TRs from T2WI and CE-T1WI, and for combined intratumoral and peritumoral regions (T-PTRs). A fusion model integrated optimal T-PTRs features from both sequences. Model performance was evaluated using the area under the receiver operating characteristic curve (AUC).

**Results:**

The T-PTRs radiomics models outperformed the TR models, with the T-PTRs (3 mm) model demonstrating optimal performance. The integration of T2WI and CE-T1WI further enhanced the T-PTRs model, yielding an AUC of 0.892(0.814-0.969) on the internal test set and an AUC of 0.828(0.741 - 0.915) on the external validation set.

**Conclusion:**

The predictive model based on intratumoral and peritumoral radiomics features serves as a valuable tool for predicting the therapeutic efficacy of HIFU ablation of uterine fibroids.

## 1 Introduction

Histologically normal tissues adjacent to tumors has long been regarded as a healthy control in cancer research, but this view overlooks the fact that the surrounding tissue is actually in a special state between healthy tissue and the tumor, with molecular and cellular composition characteristics different from those of healthy normal tissue (1). With the deepening of tumor biology research, the influence of peritumoral tissue on tumor treatment efficacy has gradually become a key direction in clinical research (2–4). However, recent studies have shown that peritumoral tissue have molecular and cellular composition characteristics that differ from healthy normal tissues, not only potentially contributing to tumor initiation but also playing a significant role in therapeutic response (5, 6).

Uterine fibroids are the most common benign gynecological tumors worldwide, affecting approximately 70% -80% of women of childbearing age and subsequently impacting their quality of life (7–9). High intensity focused ultrasound (HIFU), as an innovative non-invasive treatment technique, has been proven to effectively alleviate symptoms of uterine fibroids while preserving fertility (10, 11). However, some patients find it difficult to achieve optimal treatment outcomes under standardized treatment protocols. Researches have indicated that these differences are associated with the heterogeneity of uterine fibroid lesions. Specifically, the differences in treatment efficacy are closely related to factors such as the size, location, degeneration, and subtype of uterine fibroids (12, 13). During HIFU treatment, the therapeutic focus is primarily directed at the uterine fibroid lesion. However, as the medium through which sound waves propagate, the peritumoral tissue may change the propagation path of sound waves, absorb or scatter some energy, resulting in a decrease in the actual sound energy received by the target area, affecting the rate of temperature rise and the degree of coagulative necrosis in the lesion area. The aforementioned mechanism indicates that peritumoral tissue is not merely a passive acoustic channel, its properties play a crucial regulatory role in HIFU energy transmission. Therefore, in-depth research on the influence of surrounding tissues of uterine fibroids on the effectiveness of HIFU treatment can help optimize treatment parameters and improve treatment success rates. Currently, research in this area remains scarce, and systematically exploring the influence of peritumoral tissue on treatment outcomes will be one of the key directions for future optimization of HIFU therapy.

To enhance the efficacy of HIFU therapy and achieve precise treatment, numerous studies have attempted to classify uterine fibroid lesions based on radiological features derived from multiparametric MRI, as well as to predict HIFU treatment efficacy using radiomics models (14–17). There is an evident link between the variability of uterine fibroid imaging appearance and the heterogeneity of histological presentation (18). The anatomical morphology, size, and location of uterine fibroids can be visualized through T2-weighted imaging (T2WI), while combined contrast-enhanced T1-weighted imaging (CE-T1WI) can reveal the blood supply of the fibroid lesions (19, 20). Typically, uterine fibroids with high signal intensity on T2WI and CE-T1WI scans are generally considered challenging for successful ablation (21–23). Additionally, studies have shown that radiomics features extracted from T2WI can effectively characterize the cellular water content, fibrosis degree, and degeneration of uterine fibroids, while radiomics features extracted from CE-T1WI can reflect the blood supply (16, 24). Although these methods assess tissue heterogeneity within the fibroid lesion, it does not fully identify the heterogeneity of the peritumoral tissue and its impact on HIFU ablation efficacy, making it challenging to further enhance the accuracy of HIFU treatment efficacy assessment. Previous studies have explored the importance of peritumoral tissue in predicting the prognosis of gastric and breast cancer patients undergoing neoadjuvant chemotherapy using radiomics, with AUC improvements of 4% and 11%, respectively (25, 26). Therefore, this study aims to explore the value of peritumoral radiomics in preoperative prediction of the efficacy of HIFU ablation for uterine fibroids.

## 2 Material and methods

### 2.1 Patients

**Figure 1:**
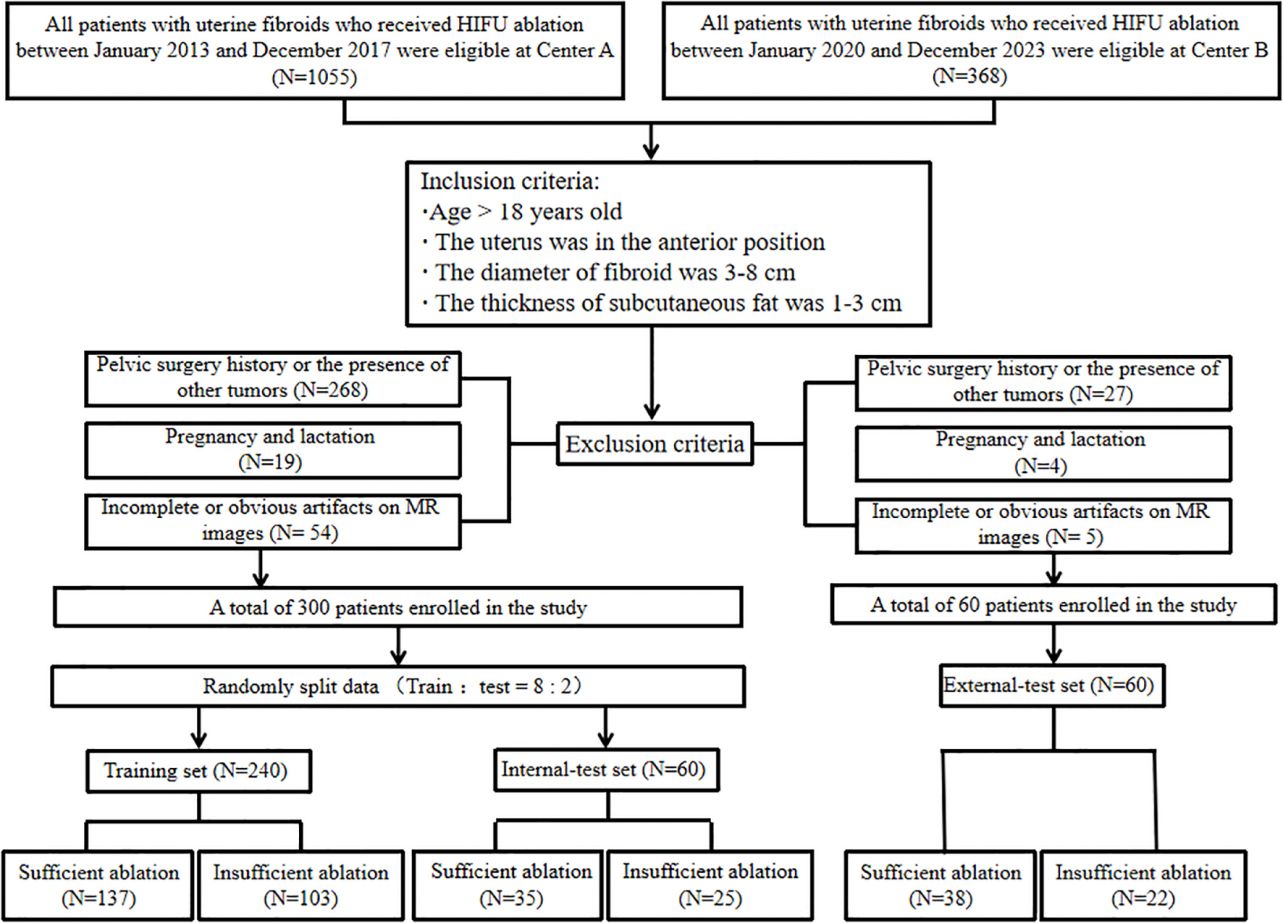
Flowchart of patient enrollment and exclusion.

The cohort included 1,055 patients who underwent HIFU treatment for uterine fibroids at Center A between January 2013 and December 2017, and an additional 288 patients at Center B from January 2020 to December 2023. The therapeutic efficacy of HIFU ablation for uterine fibroids is influenced by various factors such as fibroid size, location, tissue composition, abdominal wall thickness, and the presence of abdominal scars (36). To minimize the impact of these variables on treatment efficacy, the inclusion and exclusion criteria were established. The inclusion criteria were as follows (1) age >18 years; (2) premenopausal or perimenopausal women; (3) no relevant history of surgery or medication; (4) women who are not in the menstrual phase; (5) anterior uterine position; (6) fibroids with a diameter of 3–8 cm; (7) abdominal fat thickness of 1–3 cm; (8) for multiple fibroids, only the fibroid with the largest volume was studied; (9) Patients had pelvic MRI scans within one week before and after treatment. The exclusion criteria were as follows: (1) history of pelvic surgery or comorbidities with other tumors; (2) pregnant and lactating women; and (3) obvious artifacts in the images. Previous studies have indicated that patients with an NPVR of 80% experience significant symptom relief and a low recurrence rate (27). Thus, in this study, an NPVR ≥80% was classified as the sufficient ablation group, while an NPVR <80% was classified as the insufficient ablation group. The NPVR was independently assessed by two radiologists with differing levels of experience (4 years and 15 years in diagnostic radiology). In the case of discrepancies between the two assessments, the results of the more experienced radiologist were used as the final classification. A detailed flowchart of the enrollment process is shown in **Figure 1**. The method of HIFU treatment can be found in **Supplementary Material S1**. This retrospective study was approved by the Institutional Review Board, with a waiver for written informed consent.

### 2.2 Image acquisition and evaluation

MRI scans were acquired from two centers: one using a 3.0 T Signa HDxt MRI scanner and the other using a 1.5 T Signa Voyager MRI scanner, both provided by General Electric. The patient was placed in a supine position and underwent pelvic scanning using a dedicated 8-channel phased array coil. The protocol for conventional three-phase-enhanced MRI was to scan arterial phase images at 20s after injection of GA-DTPA (0.1 mmol/kg, 2.0 mL/s), then scanning the venous phase image 30s later, and then scanning the delayed phase image 60s later. Detailed MRI acquisition parameters are shown in **Table 1**.

**Table 1:** Detailed MRI acquisition parameters.

Parameters	Signa HDxt		Signa Voyager	
	T2WI	CE-T1WI	T2WI	CE-T1WI
Magnetic field strength	3.0 T		1.5 T	
Repetition time (TR)	270	3.84	4000	6
Echo time (TE)	2.1	1.81	68	2
Feld of view (FOV)	98.1×38	68.4×26.5	60×60	60×60
Slice thickness (mm)/gap(mm)	6/2	4/2	6/2	4/2
matrix	512×512	512×512	256x256	256x256

### 2.3 Manual segmentation and peritumoral dilation

**Figure 2:**
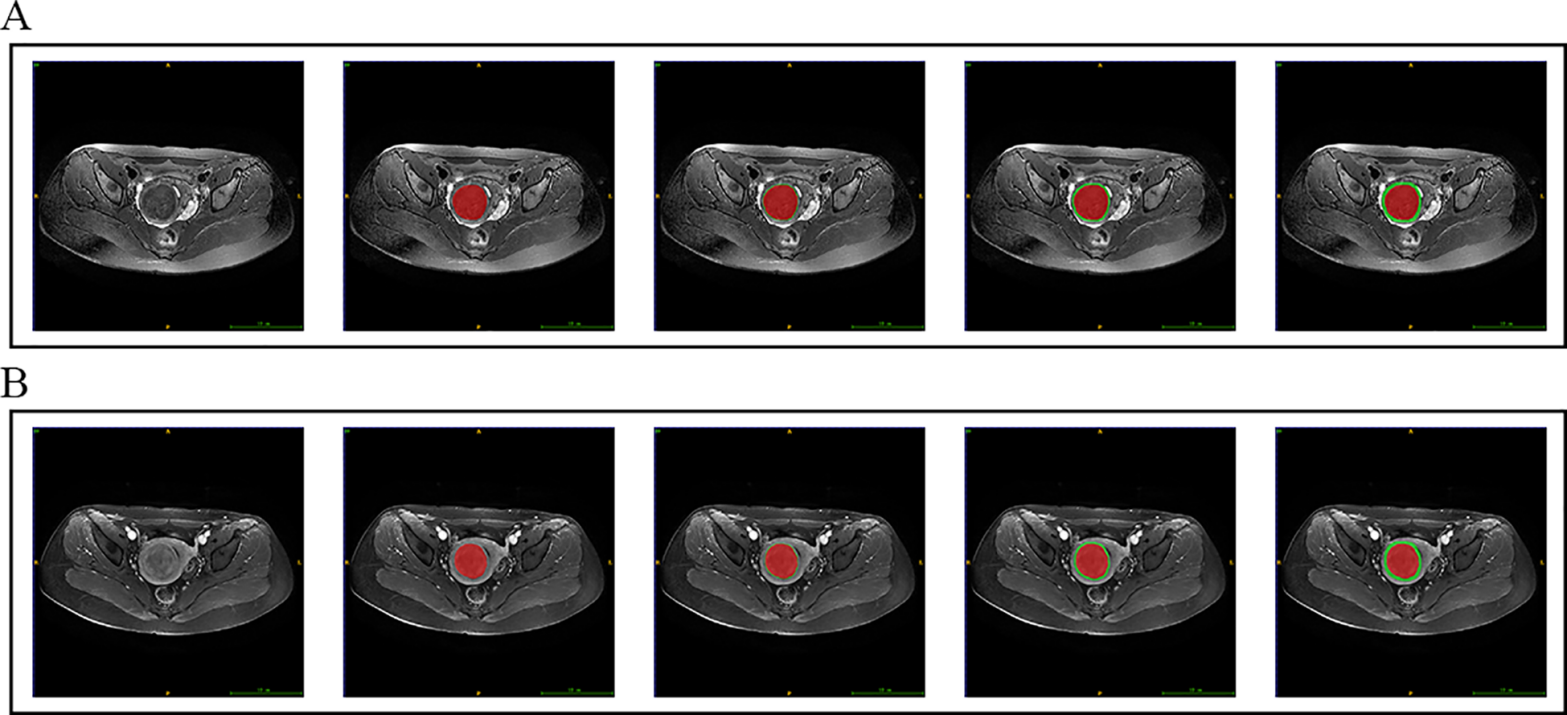
Schematic diagram of the peritumoral expansion region of a uterine fibroid. Subfigures **(A, B)** depict the peritumoral regions on T2WI and CE-T1WI, respectively, for a 35-year-old patient diagnosed with a uterine fibroid. The blue/green/yellow regions correspond to 1mm/3mm/5mm peritumoral regions (PTRs), respectively.

To delineate the boundaries of uterine fibroids in T2WI and CE-T1WI sequences and perform peritumoral region dilation, this study involved manual segmentation of MRI data from 360 patients. All MRI scans were first resampled to an isotropic voxel space of 1×1×1 mm³ using linear interpolation. Subsequently, a radiologist manually outlined the T2WI and CE-T1WI intratumoral regions (TRs) using ITK-SNAP software (version 3.8), followed by a senior radiologist who reviewed and corrected the boundaries to ensure annotation accuracy. A 3D box kernel was convolved with the original tumor regions of interest (ROIs) to maintain spatial consistency. Multiscale dilation was then implemented using the SimpleITK package in Python, applying binary morphological dilation with a spherical structuring element to expand the tumor boundary outward by 1mm, 3mm, and 5mm, generating three peritumoral regions (PTRs). The expanded PTRs were jointly validated by two radiologists, who excluded anatomical overlap with adjacent organs (e.g., intestines, bladder) and corrected discontinuous dilation caused by irregular tumor boundaries. **Figure 2** illustrates the results of manual segmentation and multi-scale dilation with manual correction.

### 2.4 Extraction of radiomics features

After all ROIs were delineated, intratumoral and peritumoral raiomics features were automatically extracted using Python. Image preprocessing methods included the original image, wavelet transform, Laplacian of Gaussian transform (LoG), square, square root, logarithm, exponential, and gradient. Radiomics features were divided into low-dimensional and high-dimensional features. Low-dimensional features included shape features and first-order histogram features, while high-dimensional features consisted of texture features: gray-level co-occurrence matrix (GLCM), gray-level run length matrix (GLRLM), gray-level size zone matrix (GLSZM), neighborhood gray-tone difference matrix (NGTDM), gray-level dependence matrix (GLDM), as well as features obtained from the texture matrix in the Gaussian Laplace filtered domain (2.0–5.0 mm kernel) and from the texture matrix in the wavelet filtered domain.

### 2.5 Intratumoral and peritumoral radiomics feature selection

A sequential feature selection process was performed on the training set to identify radiomics features related to HIFU treatment efficacy while minimizing redundancy. Both intratumoral features and the combined features from intratumoral and peritumoral regions were subject to independent selection. Interclass correlation coefficient (ICC) were used to assess the reproducibility of each radiomics feature extracted from 30 randomly chosen patients. Features with ICC values greater than 0.80 for both interobserver and intraobserver reproducibility were considered robust and selected for further analysis. Univariate analysis using Student’s t-test (p < 0.05) was performed to identify significant features across both sets of regions. To address multicollinearity, the Pearson correlations were calculated, and highly correlated features (r > 0.8) were reduced by retaining the feature with the stronger univariate association. The refined feature set was then processed using least absolute shrinkage and selection operator (LASSO) regression with 10-fold cross-validation. The optimal penalty parameter λ was selected to minimize binomial deviance, resulting in a compact set of non-redundant features.

### 2.6 Model establishment

**Figure 3:**
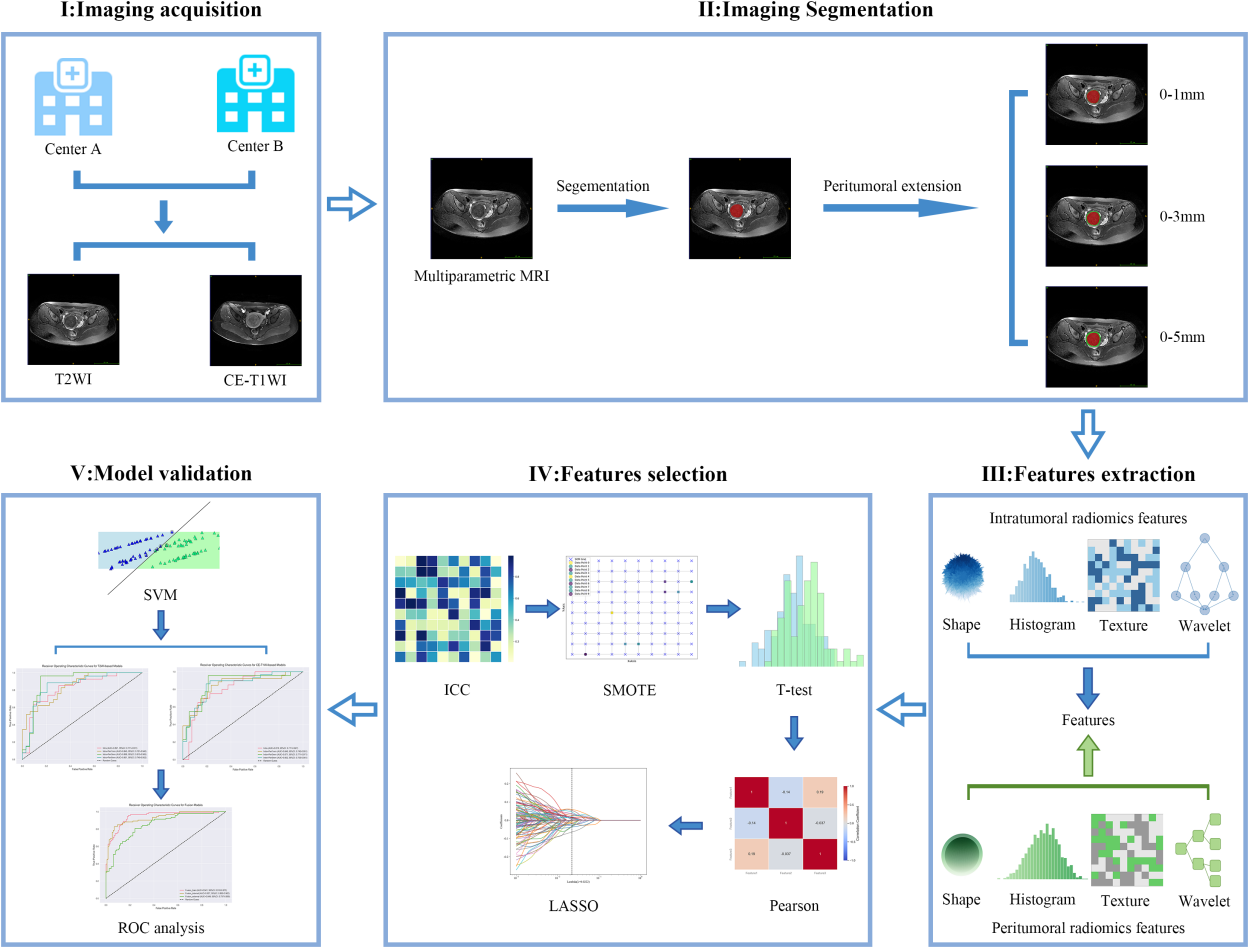
Overview of the construction workflow for the intratumoral and peritumoral prediction models.

To address class imbalance in the training dataset, the Synthetic Minority Over-sampling Technique (SMOTE) was implemented (28). The predictive model employed a support vector machine (SVM) with a radial basis function kernel, where hyperparameter optimization was conducted via grid search. Nine predictive models were developed, consisting of four T2WI-based models (TRs; TRs + 1mm PTRs; TRs + 3mm PTRs; TRs + 5mm PTRs), four CE-T1WI-based models with identical regional combinations. Subsequently, the best-performing models from both sequences were selected and used to construct an integration model of intratumoral and peritumoral regions (T-PTRs). Model performance was evaluated using the area under the receiver operating characteristic curve (AUC), with complementary assessment of accuracy, sensitivity, specificity, and F1-score. Statistical comparisons between different models were performed using the Delong test for AUC values. The radiomics analysis pipeline is shown in **Figure 3**.

### 2.7 Statistical analysis

Inter-rater reliability for radiologist assessments of the grouped results was quantified using Cohen’s kappa coefficient, with interpretation thresholds established as follows: 0.80-1.00 indicated excellent agreement, 0.40-0.79 represented moderate agreement, and values below 0.40 suggested poor agreement. Continuous variables were expressed as mean ± standard deviation, with between-group comparisons conducted using appropriate parametric (Student’s t-test) or non-parametric (Mann-Whitney U test) methods based on data distribution characteristics. Categorical variables were presented as counts and proportions, analyzed using either chi-square tests or Fisher’s exact tests depending on expected cell frequencies. A P value < 0.05 was considered statistically significant. Analyses were performed using SPSS (version 26.0, IBM) complemented by Python-based statistical packages.

## 3 Results

### 3.1 Baseline characteristics

In the study, a total of 354 patients were initially enrolled at Center A. Out of these, 54 patients were excluded due to either incomplete or obvious artifact imaging data. The remaining cohort comprised 172 patients with a favorable outcome and 128 patients with an unfavorable outcome. At Center B, 65 patients were screened, with 5 exclusions for the same reasons. The study cohort was divided into three groups: a training set (N = 240), an internal validation set (N = 60), and an external validation set (N = 60). Inter-rater agreement between the two radiologists was evaluated using Cohen’s kappa statistic. The analysis demonstrated excellent consistency, with a kappa value of 0.910 (P < 0.001). The baseline clinical and radiological features presented in **Table 2** show significant differences in fibroid type and CE signal intensity within both the training set and the internal test set.

**Table 2:** Clinical and radiological characteristics of patients.

Characteristics	Traning set (n = 240)		P value	Internal test set (N=60)		P value	External test set (N=60)		P value
	NPVR≥80% (n=137)	NPVR<80% (n=103)		NPVR≥80% (n=35)	NPVR<80% (n=25)		NPVR≥80% (n=38) NPVR<80% (n=25)	NPV<80% (n=22) NPVR<80% (n=25)	
Age (years)	39.69±6.03	38.32±6.39	0.07	39.46±7.42	42.39±5.31	0.105	38.18±8.43	40.47±7.67	0.287
Abdominal Fat Thickness (mm)	16.00±7.49	17.05±9.74	0.178	15.97±6.25	17.35±4.93	0.274	20.44±8.71	18.84±5.94	0.690
Fibroid Type			<0.001			0.029			0.165
Submucosal	3(2.22)	4(3.81)		1(2.70)	0(0)		0(0)	3(7.89)	
Intramural	115(85.19)	59(56.19)		32(86.49)	14(60.87)		18(81.82)	23(60.53)	
Subserosal	17(12.59)	42(40.00)		4(10.81)	9(39.13)		4(18.18)	12(31.58)	
T2WI Signal Intensity			0.780			0.695			0.984
Hypointensity	71(52.59)	60(57.14)		18(48.65)	9(39.13)		17(77.27)	29(76.32)	
Isointensity	21(15.56)	15(14.29)		8(21.62)	7(30.43)		2(9.09)	4(10.53)	
Hyperintensity	43(31.85)	30(28.57)		11(29.73)	7(30.43)		3(13.64)	5(13.16)	
T2WI Signal Homogeneity			0.100			0.940			0.891
Homogeneous	91(67.41)	59(56.19)		27(72.97)	16(69.57)		10(45.45)	16(42.11)	
Inhomogeneous	44(32.59)	46(43.81)		10(27.03)	7(30.43)		12(54.55)	22(57.89)	
CE-T1WI Signal Intensity			<0.001			0.004			0.359
Hypointensity	64(47.41)	77(73.33)		24(64.86)	14(60.87)		8(36.36)	21(55.26)	
Isointensity	53(39.26)	0(0)		9(24.32)	0(0)		8(36.36)	9(23.68)	
Hyperintensity	18(13.33)	28(26.67)		4(10.81)	9(39.13)		6(27.27)	8(21.05)	
CE-T1WISignal Homogeneity			0.898			0.562			0.816
Homogeneous	49(36.30)	38(36.19)		9(24.32)	8(34.78)		6(27.27)	8(21.05)	
Inhomogeneous	86(63.70)	67(63.81)		28(75.68)	15(65.22)		16(72.73)	30(78.95)	

### 3.2 Feature selection

**Figure 4:**
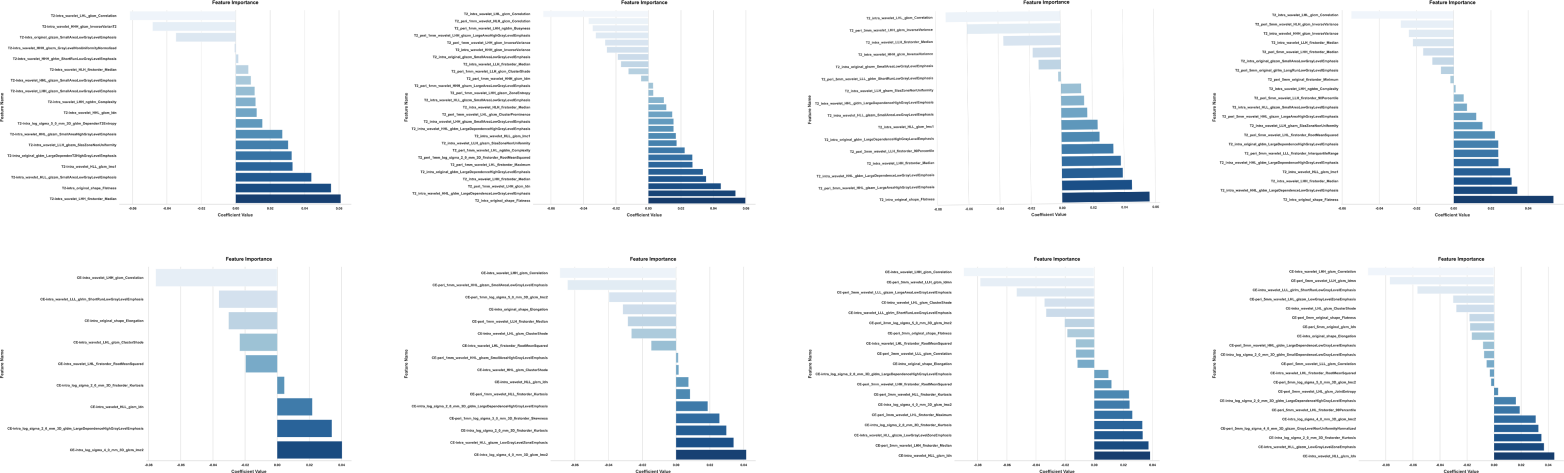
Schematic diagram of the feature importance of intratumoral and intratumoral-peritumoral radiomics features retained on T2WI and CE-T1WI.

A total of 1197 features were extracted from the intratumoral and peritumoral regions separately. After ICC consistency analysis, the ICC value of 4 feature in T2WI was less than 0.8, and the ICC value of 6 features in CE-T1WI was less than 0.8, and they were eliminated. The three-step feature selection process was employed to identify optimal radiomics features from an initial set of 1,197 extracted features per region in both T2WI and CE-T1WI sequences. For the T2WI-derived features, the final selected features comprised 18 from the intratumoral region, 27 from the TRs+1mm PTRs, 19 from the TRs+3mm PTRs, and 21 from the TRs+5mm PTRs combinations. For the CE-T1WI-derived features, a total of 9 features were retained from the intratumoral region, 16 from the TRs+1mm PTRs, 19 from the TRs+3mm PTRs, and 21 from the TRs+5mm PTRs. The detailed results of the feature selection process are provided in **Figure 4**.

### 3.3 Evaluation of model performance

**Figure 5:**
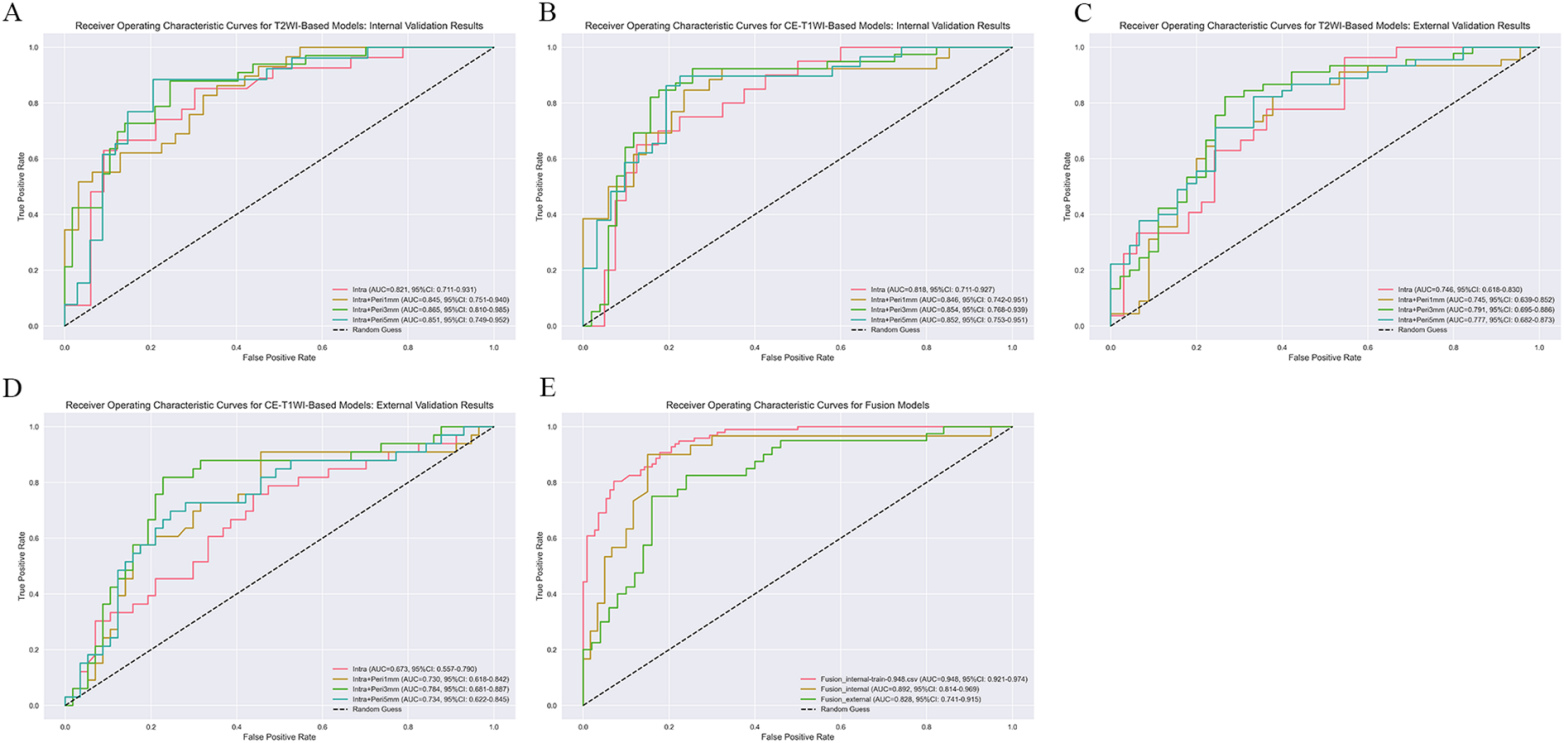
Comparing the AUCs of different models. Subfigures **(A)** and **(B)** display the AUC curves of T2WI-based and CE-T1WI-based models on the internal validation sets, respectively. Subfigures **(C)** and **(D)** show the AUC curves of T2WI-based and CE-T1WI-based models on the external validation sets, respectively. Subfigure **(E)** illustrates the AUC of the fusion model.

Performance analysis revealed that the single-modal models based on the TRs+3mm PTRs demonstrated the best performance. Specifically, the T2WI TRs+3mm PTRs model achieved an AUC of 0.865 (95%: 0.790 - 0.941) on the internal test set and 0.791 (95% CI: 0.695–0.886) on the external validation set. Similarly, the CE-T1WI TRs+3mm PTRs model yielded an AUC of 0.854(95%: 0.768-0.939) on the internal test set and 0.784 (95% CI: 0.681–0.887) on the external validation set. The fusion model, which integrated the optimal T2WI and CE-T1WI TRs+3mm PTRs features, further enhanced predictive performance, achieving an AUC of 0.892 (95% CI: 0.814-0.969) in the test set, and an AUC of 0.828(95% CI: 0.741 - 0.915) in the external validation set. Complete performance metrics, including accuracy, sensitivity, specificity, and positive predictive value for all models, are provided in **Table 3**, with comparative AUC curves shown in **Figure 5**.

**Table 3:** Performance metrics of different models.

Model	Comparison		AUC (95%CI)	Accuracy	Sensitivity	Specificity	Precision	P value
T2WI	Intra	Training set	0.875(0.829-0.920)	0.821	0.743	0.878	0.743	0.067
		Internal validation set	0.821(0.711-0.931)	0.750	0.815	0.697	0.815	0.042
		External validation set	0.726(618-834)	0.711	0.657	0.745	0.622	0.035
	Intra+Peri 1mm	Training set	0.893(0.852-0.934)	0.829	0.747	0.887	0.822	0.038
		Internal validation set	0.845(0.751-0.940)	0.733	0.828	0.645	0.686	0.045
		External validation set	0.745(0.639-0.852)	0.722	0.689	0.756	0.738	0.012
	Intra+Peri 3mm	Training set	0.881(0.836 - 0.925)	0.805	0.832	0.783	0.760	0.489
		Internal validation set	0.865(0.790 - 0.941)	0.789	0.848	0.754	0.667	0.368
		External validation set	0.791(0.695-0.886)	0.767	0.800	0.733	0.750	0.026
	Intra+Peri 5mm	Training set	0.902(0.865-0.939)	0.817	0.804	0.826	0.774	0.272
		Internal validation set	0.851(0.749-0.952)	0.801	0.846	0.794	0.749	0.042
		External validation set	0.777(0.682-0.873)	0.733	0.800	0.667	0.706	0.412
CE-T1WI	Intra	Training set	0.829(0.777-0.882)	0.775	0.722	0.818	0.765	0.018
		Internal validation set	0.818(0.711-0.927)	0.783	0.600	0.875	0.706	0.042
		External validation set	0.673(0.557-0.790)	0.622	0.727	0.561	0.490	0.072
	Intra+Peri 1mm	Training set	0.875(0.827-0.922)	0.821	0.833	0.812	0.766	0.298
		Internal validation set	0.846(0.742-0.951)	0.783	0.808	0.762	0.724	0.124
		External validation set	0.730(0.618-0.842)	0.667	0.879	0.544	0.527	0.069
	Intra+Peri 3mm	Training set	0.889(0.849-0.928)	0.812	0.745	0.866	0.814	0.027
		Internal validation set	0.854(0.768-0.939)	0.822	0.821	0.824	0.7800	0.045
		External validation set	0.784(0.681-0.887)	0.778	0.788	0.772	0.667	0.032
	Intra+Peri 5mm	Training set	0.884(0.839-0.928)	0.821	0.859	0.794	0.746	0.287
		Internal validation set	0.852(0.753-0.951)	0.817	0.862	0.774	0.781	0.036
		External validation set	0.734(0.622-0.845)	0.722	0.667	0.754	0.611	0.042
T2WI + CE-T1WI	Intra+Peri 3mm	Training set	0.948(0.921 - 0.974)	0.866	0.794	0.929	0.906	/
		Internal validation set	0.892(0.814-0.969)	0.866	0.794	0.929	0.906	/
		External validation set	0.828(0.741 - 0.915)	0.789	0.725	0.840	0.784	/

P values were obtained by performing DeLong test between T2WI + CE-T1WI (Intra+peri 3mm) model and base models constructed.

## 4 Discussion

This study developed models to predict the efficacy of HIFU ablation for uterine fibroids based on intratumoral and multiregion peritumoral radiomics features extracted from multiparametric MRI, and validated with external center data. The study results indicate that models incorporating both intratumoral and peritumoral features outperformed models based solely on intratumoral features in both MRI sequences (T2WI AUC: 0.821, CE-T1WI AUC: 0.818). Optimal predictive performance was achieved with a peritumoral extension range of 3 mm (T2WI AUC: 0.865, CE-T1WI AUC: 0.854). Notably, the fusion model further enhanced predictive performance (AUC = 0.892) by combining optimal features from both MRI sequences. It suggests that the internal regions of fibroids and surrounding tissues may contain complementary prognostic information. Comprehensive consideration of both the internal and surrounding tissues of uterine fibroids is crucial for ensuring the efficacy of HIFU ablation.

During HIFU treatment, although the primary focus is typically on the pathological region of the uterine fibroid, the characteristics of the surrounding tissue also play a significant role in the treatment outcome. Preoperative MRI provides a reliable framework for the location and shape of the fibroid; however, it primarily displays the external morphology of the fibroid and cannot comprehensively reveal the biological characteristics of both the tumor and its surrounding tissues. As tumor cells in different regions may absorb and conduct HIFU energy differently, the tumor region delineated by MRI often does not fully correspond to the biological target of HIFU ablation. Biological characteristics of the surrounding tissue, such as tissue density and blood supply, can affect the ultrasound wave propagation path and energy absorption, thereby altering the treatment efficacy to some extent. Previous studies have demonstrated that surrounding tissues, by scattering or absorbing sound waves, can hinder the effective transmission of ultrasound energy, resulting in inadequate energy accumulation within the tumor and subsequently affecting the ablation outcome (29). Therefore, the surrounding tissue is not only a medium for the transmission of HIFU energy but also plays an important regulatory role in the treatment process. To more accurately predict the effectiveness of HIFU treatment, exploring the integration of peritumoral radiomics features into existing predictive models is a promising approach. By characterizing the heterogeneity of the surrounding tissue from multiple dimensions, a better understanding of the biological behavior of the surrounding tissue can be achieved. Additionally, this approach may provide important insights for predicting and optimizing the HIFU treatment outcome.

The results demonstrate that incorporating peritumoral radiomics features into the existing framework further enhances the performance of predictive models. Different types of radiomics features can comprehensively characterize the imaging heterogeneity of peritumoral tissue surrounding uterine fibroids from multiple dimensions, including morphology, gray-level distribution, spatial relationships, and texture complexity (30, 31). This provides critical evidence for assessing their biological behavior. In the T2WI model, the wavelet_LHH_glcm_InverseVariance feature consistently persists across different tumor margin expansion ranges. This feature originates from the GLCM, reflecting local stability in image grayscale and closely correlating with the microstructural homogeneity of peritumoral tissue. Specifically, the edematous zone surrounding the fibroid exhibits high signal intensity on T2WI, a phenomenon typically associated with extracellular fluid accumulation due to local inflammatory response or increased vascular permeability. The edematous regions impair the effective transmission of ultrasound energy, resulting in uneven energy distribution and consequently affecting ablation efficacy (29, 32). Furthermore, activated fibroblasts surrounding uterine fibroids secrete substantial amounts of type I/III collagen, enhancing ultrasound energy scattering and absorption, thereby increasing the energy required for coagulative necrosis in the lesion (33). Therefore, precisely identifying changes in local grayscale stability is critical for elucidating these microscopic pathological changes, thereby enhancing the accuracy of predictive models. Concurrently, texture features extracted from CE-T1WI, by analyzing the spatial patterns of gray-level distribution, can reveal the heterogeneity of blood supply in the peritumoral tissue surrounding uterine fibroids. The heterogeneity in gray-level distribution within the imaging is often associated with uneven local blood perfusion, making texture features a valuable indirect indicator of blood flow disparities (34, 35). In addition to texture features, shape features and first-order statistical features are also retained. The morphology and vascularity of fibroids are closely interrelated, with irregular shapes or pronounced edge irregularities typically indicating greater tissue heterogeneity and potentially uneven blood supply. Particularly in the marginal regions of fibroids, neovascularization directly influences thermal diffusion during HIFU treatment, where abundant blood supply accelerates heat dissipation, thereby reducing thermal deposition (36). Meanwhile, first-order statistical features, which quantify the gray-level distribution within the image, provide complementary evidence for evaluating the vascular heterogeneity of peritumoral tissue. Notably, a fusion model integrating the optimal intratumoral and peritumoral radiomic features derived from T2WI and CE-T1WI demonstrates superior predictive performance compared to models based solely on intratumoral features. This multiparametric integration strategy leverages the complementary strengths of different MRI sequences in characterizing tissue structure and perfusion, enabling a comprehensive representation of heterogeneity in both the fibroid lesion and surrounding peritumoral tissue. Such an approach facilitates a paradigm shift in HIFU treatment, moving from traditional “morphology-based ablation” to “precision therapy guided by tissue heterogeneity.” This advancement enhances therapeutic outcomes, supports the development of individualized treatment plans, and optimizes comprehensive patient management.

This study systematically evaluated the predictive value of radiomics features derived from different peritumoral regions (1 mm, 3 mm, and 5 mm) in assessing the efficacy of HIFU ablation. The TRs+3 mm peritumoral region model demonstrated superior performance, highlighting the significant impact of peritumoral region size on radiomics analysis outcomes. Different peritumoral regions correspond to distinct tumor-host tissue interfaces, each characterized by unique tissue compositions, physiological states, and pathological responses, which may lead to significant variations in HIFU treatment outcomes. The 1 mm peritumoral region, located immediately adjacent to the fibroid boundary, is constrained by its narrow anatomical scope, making its radiomics features susceptible to partial volume effects. These features primarily reflect information related to the fibroid capsule, such as localized edema and fibrous disruption, but may fail to capture wider tissue interactions. In contrast, the 5 mm peritumoral region encompasses a broader area, potentially extending into relatively normal or unaffected myometrial tissue. Consequently, its radiomics features are often diluted by nonspecific information from the uniform texture of normal myometrium, weakening the pathological signals directly related to treatment, such as inflammatory infiltration or abnormal perfusion, and reducing predictive performance and clinical interpretability. The 3 mm peritumoral region, however, provides an optimal spatial range, focusing on the active transitional zone at the tumor invasion front, which exhibits significant biological heterogeneity. This region not only captures dynamic pathological processes, such as tissue regeneration, immune infiltration, and stromal remodeling, but also encompasses critical anatomical areas influencing HIFU energy transmission efficiency, including ultrasound propagation, scattering, and absorption. Therefore, the 3 mm peritumoral region offers superior discriminative power and clinical relevance in predicting HIFU treatment outcomes.

Our research has several limitations. Firstly, this is a retrospective study that requires larger prospective data to validate the performance of the model. Secondly, the efficacy of HIFU treatment for uterine fibroids is influenced by various factors. Therefore, future research should incorporate additional clinical indicators to further improve the accuracy and reliability of the model.

## 5 Conclusion

In conclusion, peritumoral radiomics features effectively capture the surrounding tissue characteristics of uterine fibroids, providing strong predictive value for preoperative assessment of HIFU ablation efficacy. The integrated multiparametric MRI model, combining intratumoral and optimized peritumoral features, delivers excellent preoperative prediction performance, offering a robust basis for personalized treatment planning.

## Data Availability

The raw data supporting the conclusions of this article will be made available by the authors, without undue reservation.
