# Supplementary material for: Intratumoral and peritumoral radiomics for preoperative prediction of the efficacy of HIFU ablation for uterine fibroids based on multiparametric MRI: a multicenter study
Source: Front Oncol. 2026 Jan 13;15:1699632. doi: 10.3389/fonc.2025.1699632 (PMC12834714; doi:10.3389/fonc.2025.1699632)
Supplement: Supplementary file 1 [file DataSheet1.docx]

**HIFU Treatment**

Both centers employed HIFU ablation using the JC-type focused ultrasound tumor therapeutic system (Chongqing Haifu Medical Technology Co., Ltd., Chongqing, China), with real-time monitoring conducted via a color Doppler ultrasound scanner (Mylab 70, Esaote, Italy). The therapeutic ultrasound transducer operated at a frequency of 0.8 MHz, had a diameter of 20 cm, a maximum power output of 400 W, and a focal region measuring 1.5 mm × 1.5 mm × 8.0 mm with a focal length of 18 cm. The onboard imaging ultrasound system operated at 3.5 MHz. During the procedure, patients were positioned in a prone stance on the treatment table, ensuring full contact between the lower abdominal skin and degassed water. The acoustic power and energy dose were adjusted based on patient tolerance and the observed grayscale changes in the fibroid on ultrasound imaging. Treatment was concluded when the grayscale alteration (echo enhancement) extended over the majority of the fibroid.
